# The relationship between mobile phone addiction and depression, anxiety among Chinese college students: the mediating role of friendship quality and the moderating effect of preference for solitude

**DOI:** 10.3389/fpsyt.2026.1859953

**Published:** 2026-07-01

**Authors:** Xin Mi, Xincheng Li

**Affiliations:** 1 School of Education, Shanghai Normal University, Shanghai, China; 2 Faculty of Education, Shaanxi Normal University, Xi’an, China

**Keywords:** anxiety, depression, friendship quality, mobile phone addiction, preference for solitude

## Abstract

**Background:**

The university stage represents a critical period for the development of individual mental health. Mobile phone addiction is closely linked to depression and anxiety among college students, and both friendship quality and preference for solitude are tightly associated with college students’ mobile phone addiction and emotional health. Therefore, this study aimed to investigate the relationships and internal mechanisms among mobile phone addiction, friendship quality, preference for solitude, depression and anxiety in college students.

**Methods:**

A total of 1083 Chinese college students (58.2% female; mean age = 19.87 ± 1.692 years) were included as participants. Data were collected using the Mobile Phone Addiction Index, Friendship Quality Questionnaire, Preference for Solitude Questionnaire, and Depression Anxiety Stress Scale. Data processing and analyses were conducted using SPSS 26.0 and the PROCESS macro.

**Results:**

(1) Mobile phone addiction was significantly negatively correlated with friendship quality, and significantly positively correlated with both depression and anxiety; friendship quality was significantly negatively correlated with depression and anxiety; preference for solitude was significantly positively correlated with depression and anxiety. (2) Mobile phone addiction not only directly and positively predicted depression and anxiety among college students, but also predicted depression and anxiety through the mediating role of friendship quality. (3) The direct effect of mobile phone addiction on depression and the mediating effect of friendship quality in the relationships between mobile phone addiction and depression/anxiety were both moderated by preference for solitude, whereas the moderating effect of preference for solitude on the association between mobile phone addiction and anxiety was not significant.

**Conclusion:**

Friendship quality serves as an important mediating pathway between mobile phone addiction and depressive and anxiety symptoms among Chinese college students. Preference for solitude may amplify the associations of mobile phone addiction with poorer friendship quality and elevated depressive symptoms.

## 1 Introduction

Smartphones have been deeply embedded in the daily study and life of college students, acting as an indispensable life medium and exerting a pivotal role in the development of college students’ psychosocial adaptation (1). Meanwhile, the easy accessibility and wide popularity of smartphones have also led to numerous potential risks in college students’ mobile phone use behavior, among which mobile phone addiction is a typical problematic behavior (2). Mobile phone addiction is a type of addictive behavior. It specifically refers to uncontrolled and excessive mobile phone use that is associated with negative behavioral manifestations and impaired physical and mental health. This problem has a relatively high incidence among college students, with a detection rate ranging from 21.4% to 27.4% (3). In recent years, the association between mobile phone addiction and psychosocial adaptation among college students has gradually received extensive attention from the academic community (4–6). In particular, the impact of mobile phone addiction on college students’ emotional health has become a key focus in relevant research fields. Empirical studies adopting network analysis further reveal that problematic smartphone use has stable and specific pathological association pathways with depression and anxiety in college students (7, 8), which highlights the practical urgency and research necessity of this issue.

### 1.1 Mobile phone addiction and depression, anxiety among college students

Depression and anxiety are important indicators for evaluating the emotional health status of college students (9). These negative emotions significantly reduce college students’ subjective life satisfaction. They also exert severe adverse impacts on their mental health development and daily behavioral adjustment (10). In view of this, numerous scholars have conducted in-depth explorations into the occurrence mechanisms and developmental pathways of depression and anxiety among college students from multidisciplinary perspectives (11–13). As the detection rate of mobile phone addiction among college students continues to rise, mobile phone addiction has gradually become a critical research entry point for analyzing the triggers of depression and anxiety in this population. Existing empirical studies have confirmed that mobile phone addiction not only impairs individuals’ sleep quality (14–16), but also correlates with academic procrastination and behavioral procrastination (17, 18). In turn, sleep disturbances, poor sleep quality, and habitual procrastination have been identified as key antecedent variables associated with depression and anxiety (19, 20). Furthermore, addictive behaviors stemming from excessive smartphone dependence tend to exacerbate individuals’ interpersonal impairment and social alienation, thereby correlating with higher levels of negative emotional experiences such as depression and anxiety (21, 22). Therefore, mobile phone addiction is correlated with an elevated risk of depression and anxiety among college students. Three-wave longitudinal studies conducted among Chinese college students further confirm that this association is not merely the concurrent correlation reported in cross-sectional studies. Instead, mobile phone addiction shows prospective correlational links with depressive and anxiety symptoms (23).

### 1.2 The mediating role of friendship quality

Previous studies have confirmed that mobile phone addiction is associated with various interpersonal adaptation disturbances, including social anxiety, interpersonal alienation, and interpersonal relationship distress (21, 24, 25). Based on this, it can be inferred that the poor interpersonal relationships correlated with mobile phone addiction may act as a critical bridge linked to the subsequent development of negative emotions such as depression and anxiety. Friendship quality is an important indicator of interpersonal relationship functioning. It is comprehensively influenced by multiple factors, including environmental conditions, individual traits, and behavioral patterns (26–28). The potential association between mobile phone addiction and friendship quality has gradually attracted academic attention and investigation. Existing research indicates that individuals with mobile phone addiction tend to overindulge in virtual social contexts and neglect traditional interpersonal interaction and emotional bonding in real-life settings, thereby exhibiting lower levels of interpersonal intimacy (29). Similarly, studies targeting college student samples have found that excessive mobile phone use occupies a large amount of offline social time, restricts the development of interpersonal skills to a certain extent, and consequently correlates with problems such as interpersonal disharmony, relationship distress, and loneliness (21, 30). Other research has demonstrated that the unregulated, indiscriminate mobile phone use characteristic of mobile phone addiction undermines individuals’ friendship quality (31). In addition, friendship quality is a significant correlate of emotional problems including depression and anxiety (32, 33). As a vital source of social support, high-quality friendship can effectively alleviate individuals’ negative emotional experiences. Existing studies have also verified that friendship quality is consistent with exerting a mediating role in the relationship between variables such as parent-child attachment and individuals’ emotional states (34). Therefore, the first hypothesis of the present study is proposed: Friendship quality plays a mediating role in the relationships between mobile phone addiction and depression, anxiety.

### 1.3 The moderating role of preference for solitude

Preference for solitude is also a critical factor influencing individuals’ friendship quality and emotional health, referring to the degree to which individuals prefer to be alone (35). Within the Chinese collectivist culture, society emphasizes interpersonal dependence and group connectedness. Solitude is often viewed as contradictory to collective values. It is even labeled as a selfish or maladaptive behavioral manifestation (36). Driven by this cultural characteristic, preference for solitude may exert negative effects on the psychosocial adaptation of Chinese college students. Relevant studies have verified that preference for solitude not only directly and positively predicts individuals’ psychological maladjustment, but also further undermines their psychological adaptation by reducing their level of social acceptance (37, 38). Meanwhile, preference for solitude is a relatively stable personality trait closely associated with psychological adjustment problems such as high shyness and social anxiety. Specifically, individuals with a high preference for solitude are more prone to negative emotions, including loneliness, depression, and anxiety (39, 40). Therefore, preference for solitude may strengthen correlational associations between mobile phone addiction and depression, anxiety. Compared with their counterparts with a low preference for solitude, individuals with a high preference for solitude are more likely to develop depression and anxiety when suffering from mobile phone addiction. In addition, as mentioned earlier, excessive mobile phone use accompanied by mobile phone addiction is a major factor correlated with declines in individuals’ friendship quality (31). Individuals with a high preference for solitude who are also addicted to mobile phones tend to neglect necessary real-life interpersonal interactions due to smartphone overindulgence. This implies that the negative impact of mobile phone addiction on friendship quality may be more pronounced among those with a high level of preference for solitude. Accordingly, the following hypotheses are proposed in this study: Preference for solitude plays a moderating role in the relationships between mobile phone addiction and friendship quality, depression, and anxiety.

Although existing studies have respectively verified the association between mobile phone addiction and emotional health, the protective role of friendship quality, and the potential influence of preference for solitude, three major limitations can be identified in current research:

First, research on underlying mechanisms remains fragmented. Most prior studies have either solely examined the direct effect of mobile phone addiction on depression and anxiety, or separately explored the role of a single mediator variable (e.g., loneliness, sleep quality) (41, 42). Few studies have taken impaired interpersonal interaction caused by mobile phone addiction as a key mediating pathway, let alone incorporated the moderating effects of personality traits simultaneously. Accordingly, the underlying mechanisms explaining the correlational patterns between mobile phone addiction and college students’ emotional health have not been fully elaborated.

Second, homogenized assumptions exist regarding moderating effects. Current research generally regards depression and anxiety as highly correlated negative emotions and assumes that a given variable exerts consistent mechanisms of action on both outcomes (43). Nevertheless, the inherent differences between the two emotions in their neural basis, cognitive processes and triggering factors have been largely overlooked. As for preference for solitude, a vital personality trait, few studies have systematically compared and clarified its differentiated moderating effects on the two pathways: mobile phone addiction → depression and mobile phone addiction → anxiety.

Third, limitations related to cultural context. Most studies on preference for solitude are conducted against the backdrop of Western individualistic cultures, where moderate solitude is considered to exert positive psychological functions (44). By contrast, in Chinese collectivistic culture that emphasizes interpersonal bonds, solitude is often perceived as a sign of maladjustment. Hence, its moderating role in the relationship between mobile phone addiction and emotional health may follow a completely different pattern.

**Figure 1:**
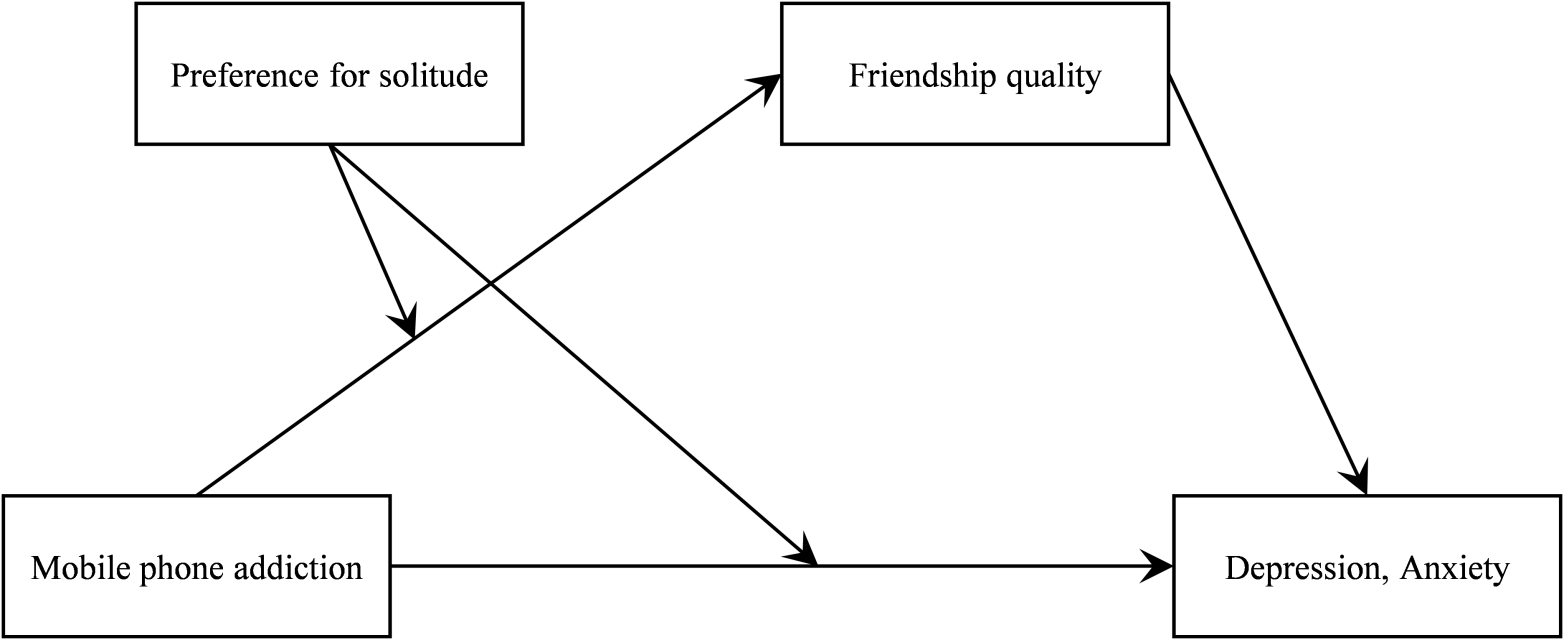
Hypothesized model.

Accordingly, this study constructed and tested an integrated hypothesized moderated mediation model to address three key theoretical gaps (**Figure 1**). First, we clarified friendship quality as a mediating pathway linking mobile phone addiction to depressive and anxiety symptoms. We specifically tested the sequential transmission pattern: mobile phone addiction → reduced offline interpersonal interaction →decreased friendship quality → elevated negative emotions. Second, we systematically examined the moderating effects of preference for solitude on all pathways in the model, with a focus on its differential impacts on depression versus anxiety. Third, we demonstrated the amplifying role of preference for solitude in the Chinese collectivistic cultural context. This study provides cross-cultural evidence for understanding the mechanisms underlying college students’ emotional problems in the digital age. The findings advance theoretical understanding of the association between mobile phone addiction and emotional health. They also provide scientific evidence for universities to develop targeted mental health interventions.

## 2 Methods

### 2.1 Participants

The present study recruited college students from four universities in Shanghai and Shaanxi Province as research participants. A questionnaire survey was administered using convenience sampling to measure participants’ levels of mobile phone addiction, friendship quality, preference for solitude, depression, and anxiety. A total of 1270 questionnaires were collected. To ensure data quality, the present study established the following rigorous exclusion criteria for invalid questionnaires:

First, exclusion criterion for abnormal response duration. Based on the distribution of response duration across the full sample (median = 6.2 minutes, interquartile range = 2.8 to 10.5 minutes), and in accordance with commonly used criteria in the field, questionnaires with a response duration of < 120 seconds or > 1200 seconds were excluded. An excessively short response duration typically suggests that participants did not respond carefully, whereas an excessively long duration may indicate that participants interrupted responding or completed the questionnaire with multiple pauses, resulting in lower data reliability.

Second, exclusion criterion for stereotyped response patterns. A questionnaire was deemed invalid if it met any of the following conditions: (1) selection of the same option for 10 or more consecutive items; (2) presence of cyclical repetitive response patterns (e.g., 1-2-3-1-2-3); (3) selection of extreme values for all items (the lowest possible score or the highest possible score on the corresponding scale, with the exception of reverse-scored items). These response patterns suggest that participants did not engage in genuine responding and constitute invalid responses.

Third, exclusion criterion for failing the attention check. The present study embedded one instructional attention check item at the position of Item 16: “To ensure that you are responding carefully to this questionnaire, please select Option 3.” Failure to select the specified option was deemed to indicate failure of the attention check. Such participants may not have carefully read the questionnaire instructions, and their data may not reflect their true psychological states.

Based on the above criteria, a total of 187 invalid questionnaires were excluded, including 92 with abnormal response duration, 65 with stereotyped response patterns, and 30 that failed the attention check. The final valid sample comprised 1083 participants, representing an effective response rate of 85.28%. Participants ranged in age from 16 to 25 years (M ± SD = 19.87 ± 1.692). Among them, 453 were male (41.8%) and 630 were female (58.2%). Regarding grade level, 276 were freshmen (25.5%), 252 were sophomores (23.3%), 270 were juniors (24.9%), and 285 were seniors (26.3%).

### 2.2 Measures

#### 2.2.1 Mobile phone addiction index

The Mobile Phone Addiction Index (MPAI) developed by Leung (3) was adopted to assess mobile phone addiction among college students. This scale consists of 17 items, all rated on a 5-point Likert scale ranging from 1 to 5. Higher total scores indicate more severe levels of mobile phone addiction. In the present study, the Cronbach’s α coefficient of the Mobile Phone Addiction Index was 0.836.

#### 2.2.2 Friendship quality questionnaire

The Chinese version of the Friendship Quality Questionnaire (FQQ) revised by Cui et al. (45) was adopted to assess participants’ friendship quality. This questionnaire consists of 14 items rated on a 7-point Likert scale ranging from 1 to 7. After reverse-scoring several items, the mean score of all items was computed to represent overall friendship quality, with higher scores indicating better friendship quality. In the present study, the Cronbach’s α coefficient of this questionnaire was 0.889.

#### 2.2.3 Preference for solitude questionnaire

The Chinese version of the Preference for Solitude Questionnaire, originally developed by Burger (39) and revised by Chen and Zhou (46), was used to assess participants’ preference for solitude. This questionnaire consists of 11 items, all of which employ a forced-choice format with two mutually exclusive options for each item. Participants were required to select the option that best reflected their own situation from the two choices. Specifically, one point was awarded for each selection related to a preference for solitude, and the mean score across all items was used to represent participants’ preference for solitude, with higher scores indicating a stronger degree of preference for solitude.

In terms of psychometric properties, all items of this questionnaire adopt dichotomous scoring. Cronbach’s α is applicable to multi-point Likert-type interval scales and is not appropriate for dichotomous items. Accordingly, this study adopted the KR-20 coefficient, which is specifically designed for scales with dichotomous scoring, to evaluate internal consistency reliability. The results showed that the KR-20 coefficient of this questionnaire was 0.867. Individual forced-choice items are categorical variables and cannot be directly included in regression analysis. After calculating the mean score across all 11 items, the derived scores become quasi-continuous variables. Given the large sample size of the present study (N = 1083), these mean scores approximately satisfy the requirements for interval data. This data processing method has been widely adopted in studies using similar forced-choice scales. Therefore, scores from the Preference for Solitude Questionnaire can be combined with data from other interval scales in this study to conduct regression analysis and moderated mediation analysis.

#### 2.2.4 Depression, anxiety and stress scale

The Depression, Anxiety and Stress Scale (DASS-21) developed by Lovibond and Lovibond (47) was adopted, and only the depression and anxiety subscales were used to assess participants’ levels of depression and anxiety. All items were rated on a 4-point Likert scale ranging from 0 to 3. The mean scores of items within the depression and anxiety subscales were calculated respectively to represent participants’ scores in the corresponding dimensions, with higher scores indicating more severe levels of depression and anxiety. In the present study, the Cronbach’s α coefficients of the depression and anxiety subscales were 0.918 and 0.907, respectively.

### 2.3 Data analysis

For data analysis, SPSS 26.0 was adopted to perform descriptive statistics, correlation analysis and Harman’s single-factor common method bias testing. Amos 29.0 was used for confirmatory factor analysis and common method bias testing. The PROCESS macro version 4.0 (Model 8) developed by Hayes (48) was employed to examine the moderated mediation effect. The Bootstrap resampling method with 5,000 repetitions was performed to report the 95% confidence intervals.

## 3 Results

### 3.1 Common method bias test

This study adopted a combined approach of Harman’s single-factor test and confirmatory factor analysis (CFA) to assess the common method bias.

First, Harman’s single-factor test was conducted by performing unrotated exploratory factor analysis on all items. The results revealed that the variance explained by the first common factor was 28.62%, which was lower than the critical threshold of 40% (49). This preliminary result indicated no severe common method bias in the present research.

Second, Amos 29.0 software was utilized to conduct confirmatory factor analysis on the five-factor model consisting of mobile phone addiction, friendship quality, preference for solitude, depression and anxiety. This model was further compared with one-factor, two-factor, three-factor and four-factor models. The fit indices of the five-factor model were as follows: χ²/df = 4.217, CFI = 0.942, TLI = 0.935, SRMR = 0.043, RMSEA = 0.054. The five-factor model exhibited obviously better fitting performance than other alternative models, which verified favorable discriminant validity of the five-factor structure (see **Table 1**).

**Table 1:** Confirmatory factor analysis results.

Models	χ²/df	CFI	TLI	SRMR	RMSEA
Five-factor model + CMV	3.189	0.963	0.954	0.036	0.045
Five-factor model: MPA, FQ, PFS, DEP, ANX	4.217	0.942	0.935	0.043	0.054
Four-factor model: MPA, FQ, PFS, DEP+ANX	8.762	0.856	0.841	0.078	0.085
Three-factor model: MPA+PFS, FQ, DEP+ANX	15.328	0.742	0.721	0.102	0.115
Two-factor model: MPA+PFS+FQ, DEP+ANX	21.945	0.678	0.653	0.121	0.139
One-factor model: MPA+PFS+FQ+DEP+ANX	28.734	0.621	0.598	0.108	0.160

n, 1083; MPA, Mobile phone addiction; FQ, Friendship quality; PFS, Preference for solitude; DEP, Depression; ANX, Anxiety.

Furthermore, an additional common method variance (CMV) factor was incorporated into the original five-factor model. Model fit indices did not display marked improvement (ΔCFI=0.021, ΔTLI=0.019, ΔSRMR=0.007, ΔRMSEA=0.009). If the original confirmatory factor analysis (CFA) model with correlated factors demonstrates notable improvements in fit indices following the addition of a method factor (e.g., CFI and TLI increases exceeding 0.1, RMSEA and SRMR decreases surpassing 0.05), it suggests the presence of substantial common method bias (50). Collectively, these results indicate that common method bias is unlikely to substantially threaten the findings of this study.

### 3.2 Descriptive statistics and correlation analysis of mobile phone addiction, friendship quality, preference for solitude, depression, and anxiety

Correlation analysis results for all variables revealed that mobile phone addiction was significantly negatively correlated with friendship quality, and significantly positively correlated with depression and anxiety. Friendship quality was significantly negatively correlated with depression and anxiety. Preference for solitude was significantly positively correlated with depression and anxiety. Detailed results are presented in **Table 2**.

**Table 2:** Results of descriptive statistics and correlation analysis.

Variable	M	SD	1	2	3	4	5
1. Mobile phone addiction	2.703	0.582	1				
2. Friendship quality	5.287	0.729	-0.248**	1			
3. Preference for solitude	0.776	0.258	0.081*	0.029	1		
4. Depression	1.072	0.761	0.309**	-0.256**	0.538**	1	
5. Anxiety	1.118	0.719	0.307**	-0.221**	0.554**	0.897**	1

*p < 0.05, **p < 0.01.

### 3.3 Testing of the moderated mediation effect

After controlling for demographic variables including gender and age, Model 8 of the SPSS PROCESS macro developed by Hayes (48) was applied along with the bias-corrected percentile Bootstrap method. We examined the direct predictive effect of mobile phone addiction on depression and anxiety, further explored the mediating effect of friendship quality and moderating effect of preference for solitude within these relationships, and estimated the corresponding 95% confidence intervals.

Results of the moderated mediation analysis (see **Table 3**) showed that mobile phone addiction had a significant positive predictive effect on both depression and anxiety. After including mobile phone addiction, friendship quality, preference for solitude, and the interaction term between mobile phone addiction and preference for solitude in the moderated mediation model for regression analysis, it was found that mobile phone addiction significantly predicted friendship quality, depression, and anxiety, and friendship quality also significantly predicted depression and anxiety. Meanwhile, the 95% Bootstrap confidence intervals for the mediating effect of friendship quality did not include 0 (see **Table 4**), supporting partial mediation in the associations between mobile phone addiction and both depressive and anxiety symptoms. In addition, the interaction term between mobile phone addiction and preference for solitude significantly predicted friendship quality and depression, but had no significant predictive effect on anxiety. This indicates that preference for solitude moderated the associations between mobile phone addiction and friendship quality, and between mobile phone addiction and depression, but not the association between mobile phone addiction and anxiety. Results of the simple slope test indicated that, compared with individuals with a low preference for solitude, mobile phone addiction was more likely to reduce friendship quality among those with a high preference for solitude (simple slope for the high group = −0.292, t = −8.591, *p* < 0.001; simple slope for the low group = −0.241, t = −7.386, *p* < 0.001) and to induce depressive symptoms (simple slope for the high group = 0.293, t = 8.571, *p* < 0.001; simple slope for the low group = 0.242, t = 6.688, *p* < 0.001). The direct effect of mobile phone addiction on depression, as well as the indirect effects of friendship quality in the relationships between mobile phone addiction and depression/anxiety, were more prominent among individuals with a high preference for solitude. However, the direct effect of mobile phone addiction on anxiety was not moderated by preference for solitude (see **Table 4**).

**Table 3:** Analysis of the moderated mediation effect.

Dependent variable		Depression						Anxiety					
Outcome variable	Predictor variable	Fit Indices				Coefficient and significance		Fit indices				Coefficient and significance	
		R	R^2^	*F*	B	*β*	*t*	R	R^2^	*F*	B	*β*	*t*
Depression/Anxiety		0.372	0.138	35.892				0.377	0.144	37.611			
	Gender				0.156	0.101	4.381**				0.187	0.128	5.264**
	Age				0.117	0.260	3.276**				0.094	0.221	2.642**
	Mobile phone addiction				0.304	0.232	8.497**				0.317	0.257	8.392**
Friendship quality		0.345	0.119	21.984				0.345	0.119	21.984			
	Gender				0.211	0.143	5.371**				0.211	0.143	5.371**
	Age				-0.041	-0.095	-1.058				-0.041	-0.095	-1.058
	Mobile phone addiction				-0.261	-0.208	-8.129**				-0.261	-0.208	-8.129**
	Preference for solitude				-0.014	-0.005	-0.357				-0.014	-0.005	-0.357
	INT				-0.094	-0.019	-3.381**				-0.094	-0.019	-3.381**
Depression/Anxiety		0.651	0.422	69.983				0.648	0.417	79.872			
	Gender				0.038	0.025	1.118				0.058	0.040	1.724
	Age				0.031	0.069	1.002				0.011	0.026	0.352
	Friendship quality				-0.221	-0.212	-5.957**				-0.192	-0.195	-5.776**
	Mobile phone addiction				0.203	0.155	5.918**				0.209	0.169	6.051**
	Preference for solitude				0.517	0.175	16.698**				0.525	0.188	15.861**
	INT				0.071	0.014	2.094*				0.031	0.006	0.847

*p < 0.05, **p < 0.01. B = unstandardized coefficient, β = standardized coefficient. INT refers to the centered product term of mobile phone addiction and preference for solitude. All variables in the model were entered into the regression equation as mean scores.

**Table 4:** Moderating effect analysis of preference for solitude.

Effect type	Preference for solitude	Depression					Anxiety				
		B	Boot SE	Boot 95% CI lower	Boot 95% CI upper	β	B	Boot SE	Boot 95% CI lower	Boot 95% CI upper	β
Direct effect	M−1SD	0.128	0.048	0.032	0.228	0.098					
	M	0.201	0.033	0.136	0.271	0.154	0.208	0.033	0.141	0.277	0.168
	M+1SD	0.259	0.041	0.176	0.344	0.198					
Indirect effect	M−1SD	0.035	0.011	0.017	0.057	0.027	0.030	0.009	0.015	0.051	0.024
	M	0.055	0.011	0.037	0.079	0.042	0.049	0.009	0.031	0.072	0.040
	M+1SD	0.072	0.013	0.049	0.101	0.055	0.063	0.012	0.041	0.092	0.051

B = unstandardized coefficient, β = standardized coefficient. Since the moderating effect of preference for solitude on the relationship between mobile phone addiction and anxiety was not significant, this table only presents the direct effect of mobile phone addiction on anxiety at the mean level of preference for solitude. Boot SE, Boot 95% CI Lower and Boot 95% CI Upper represent the standard error and the lower and upper bounds of the 95% confidence interval for the indirect effect estimated by the bias-corrected percentile Bootstrap method, respectively. All values are rounded to three decimal places.

**Figure 2:**
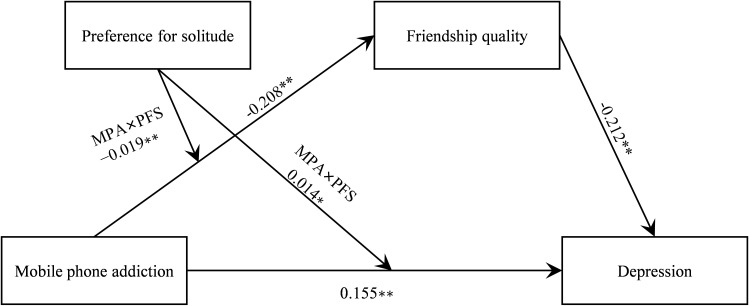
Results of the moderated mediation model for depression. *p < 0.05, **p < 0.01. MPA, Mobile phone addiction; PFS, Preference for solitude. Values are standardized regression coefficients (β). All paths were statistically significant.

To more intuitively demonstrate the results of moderated mediation effect tests and clearly present the differential moderating effects of preference for solitude on depression and anxiety, this study separately plotted path diagrams of the moderated mediation models with depression and anxiety as the dependent variables. As shown in **Figure 2**, all hypothesized paths were empirically supported in the model with depression as the dependent variable. Mobile phone addiction not only directly and positively predicts depression, but also indirectly predicts depression through the mediating role of friendship quality. Preference for solitude significantly strengthens the negative effect of mobile phone addiction on friendship quality. It also enhances the direct positive effect of mobile phone addiction on depression.

**Figure 3:**
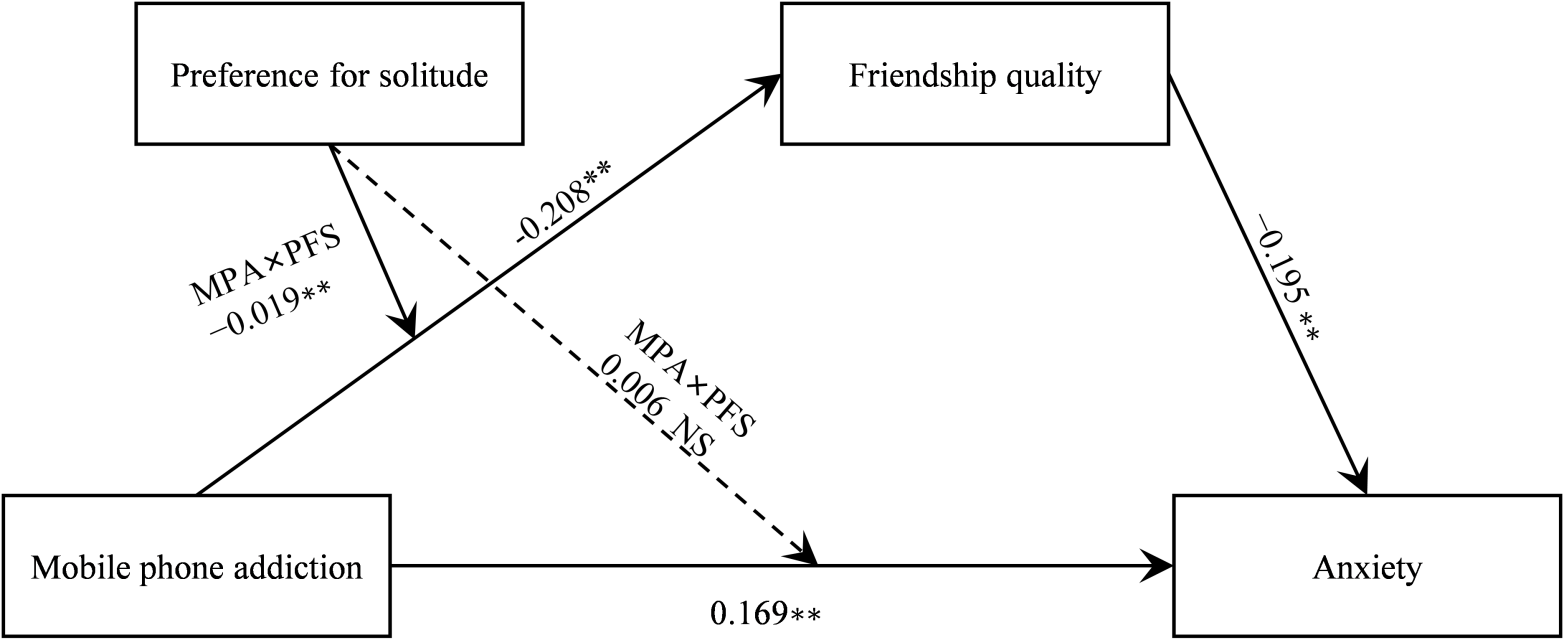
Results of the moderated mediation model for anxiety. *p < 0.05, **p < 0.01. MPA, Mobile phone addiction; PFS, Preference for solitude. NS, not significant. Values are standardized regression coefficients (β). Dashed lines indicate non-significant paths.

As shown in **Figure 3**, most hypothesized paths were empirically supported in the model with anxiety as the dependent variable. Notably, preference for solitude significantly strengthens the negative effect of mobile phone addiction on friendship quality, but exerts no significant moderating effect on the direct path from mobile phone addiction to anxiety (β=0.006, *p* > 0.05). This finding is not fully consistent with the original theoretical hypotheses of this study, suggesting differences in the moderating mechanisms of preference for solitude on depression and anxiety. Specifically, preference for solitude can simultaneously moderate both the direct effect of mobile phone addiction on depression and the indirect effect of mobile phone addiction on depression through the mediating role of friendship quality. In contrast, for anxiety, preference for solitude can only moderate the indirect effect of mobile phone addiction on anxiety through the mediating role of friendship quality, and cannot moderate the direct effect of mobile phone addiction on anxiety.

## 4 Discussion

### 4.1 Main findings

In the present study, mobile phone addiction was significantly negatively correlated with friendship quality, and significantly positively correlated with depression and anxiety. This indicates that, as a potential risk correlate of college students’ psychosocial adaptation in the digital era, mobile phone addiction is associated with interpersonal distress (51, 52), and higher levels of depression and anxiety, suggesting adverse links with college students’ emotional health (53, 54). Friendship quality was significantly negatively correlated with depression and anxiety, suggesting that high-quality friendship, as a vital source of social support for college students, correlates with better emotional health outcomes (55). Preference for solitude was significantly positively correlated with depression and anxiety, which implies that prolonged solitude may correlate with negative coping styles such as ruminative thinking among college students, and further give rise to negative emotions (56).

Results of the moderated mediation analysis revealed that mobile phone addiction not only directly and positively predicted depression and anxiety among college students, but also exhibited an indirect correlational association with these negative emotions through friendship quality. The mediating role of friendship quality reflects a clear sequential pathway. Excessive mobile phone use reduces college students’ time for face-to-face interaction and lowers the quality of these engagements. This pattern is linked to impaired friendship development among college students (21). Reduced friendship quality, in turn, corresponds to lower levels of peer social support. This correlates with elevated odds of depression and anxiety among college students. Furthermore, prolonged mobile phone use substantially undermines the development of college students’ social interaction skills (57, 58). Poor social skills make it harder to establish positive interpersonal relationships and may also correlate with heightened social anxiety symptoms to some degree. Taken together, these findings show that mobile phone addiction has indirect correlational links with college students’ depression and anxiety through reduced friendship quality.

Furthermore, the present study also found that preference for solitude moderated the relationship between mobile phone addiction and friendship quality, as well as the relationship between mobile phone addiction and depression, but did not moderate the relationship between mobile phone addiction and anxiety. In other words, the impact of mobile phone addiction on depression and the mediating effect of friendship quality in the associations between mobile phone addiction and depression/anxiety were more significant among individuals with a high preference for solitude. These findings indicate that preference for solitude may amplify the correlational patterns whereby mobile phone addiction links to poorer friendship quality and higher depressive symptoms among college students. Previous studies have shown that preference for solitude is an important factor contributing to negative emotions such as depression (59). This may be because individuals with a higher preference for solitude are more likely to adopt negative coping styles such as ruminative thinking, which may amplify correlational associations between other variables and depressive symptoms (60, 61). Therefore, compared with individuals with a low preference for solitude, those with a high preference for solitude are more prone to developing depression. On the other hand, prior research has suggested that prolonged solitude is unfavorable for the development of interpersonal friendship (62). College students with a high preference for solitude who also suffer from mobile phone addiction tend to stay alone using mobile phones, which reduces their social interaction with others to a certain extent and further lowers their friendship quality. Accordingly, compared with individuals with a low preference for solitude, mobile phone addiction is more likely to induce depression among those with a high preference for solitude by further impairing their friendship quality.

### 4.2 Theoretical explanation of asymmetric moderating effects

The most notable finding of this study is the asymmetric moderating effect of preference for solitude on the associations between mobile phone addiction and depression, and between mobile phone addiction and anxiety. It significantly strengthens the direct predictive effect of mobile phone addiction on depression, but exerts no significant moderating effect on the direct association between mobile phone addiction and anxiety. This finding can be systematically explained by two classic emotion theories in psychology.

First, the tripartite model of anxiety and depression proposed by Clark and Watson (63) provides an important theoretical rationale for this difference. This model posits that although depression and anxiety share general negative affect, they have distinct characteristic features. Depression is characterized by low positive affect (anhedonia), while anxiety is primarily marked by high physiological arousal (nervousness and restlessness). The direct effect of mobile phone addiction on depression operates primarily through the deprivation of positive affect. Mobile phone addiction displaces time that would otherwise be spent on real-world activities such as exercise and face-to-face social interaction that generate pleasure. Individuals with a high preference for solitude inherently derive less pleasure from social interaction and have more limited sources of positive affect. Accordingly, the exacerbating effect of mobile phone addiction on their depressive symptoms is significantly amplified. In contrast, the direct effect of mobile phone addiction on anxiety is rooted in high physiological arousal, such as fear of missing out on information and concerns about academic underachievement. This physiological stress response is universal across individuals and does not vary as a function of a preference for solitude, which explains why preference for solitude does not moderate this pathway.

Second, Beck’s (64) cognitive specificity theory provides a complementary explanation from the perspective of cognitive processing. This theory posits that depression and anxiety differ in their underlying cognitive content. Depression centers on “loss” (loss of self-worth and future hope), while anxiety focuses on “threat” (concerns over future danger). Mobile phone addiction is associated with negative cognitions related to self-worth loss, such as “I have wasted my time” and “I cannot control myself”. Individuals with a high preference for solitude are more prone to rumination due to a lack of positive peer feedback. They tend to generalize these negative thoughts into a global denial of self-worth, which may exacerbate depressive symptoms. In contrast, anxiety linked to mobile phone addiction is rooted in concerns about future threats. This cognitive pattern does not align with the characteristic cognitive style of individuals with a high preference for solitude, who are more likely to ruminate on past negative events. Accordingly, preference for solitude fails to amplify this effect.

It is noteworthy that preference for solitude still exerts a significant moderating effect on the mediating role of friendship quality between mobile phone addiction and anxiety. This is consistent with the theories presented above. Anxiety associated with reduced friendship quality primarily manifests as social anxiety (worries about rejection and isolation), which is an emotional state commonly experienced by individuals with a high preference for solitude. When mobile phone addiction further impairs their already fragile interpersonal relationships, social anxiety tends to be significantly amplified. This, in turn, is associated with an indirect increase in overall anxiety levels. This finding further supports the distinction between depression and anxiety and provides new empirical evidence for future research examining the distinct mechanisms underlying these two emotional states.

### 4.3 Theoretical implications

This study presents three main theoretical implications. First, this study establishes a more comprehensive model depicting the relationship between mobile phone addiction and emotional health. Most prior studies have interpreted the adverse correlational patterns of mobile phone addiction based on intrapersonal factors such as cognitive bias and emotion regulation ability (65). By further incorporating real-life interpersonal relationships, a critical social contextual factor, the present study confirms that friendship quality is consistent with acting as a vital mediating pathway linking mobile phone addiction to negative emotions. This finding broadens the theoretical perspectives for understanding how mobile phone addiction affects emotional health and provides a new analytical framework for follow-up research.

Second, this study reveals the differentiated moderating effects of preference for solitude on depression and anxiety. The results indicate that preference for solitude significantly strengthens the direct influence of mobile phone addiction on depression, as well as the indirect impacts on both depression and anxiety mediated by friendship quality. Nevertheless, its moderating role in the association between mobile phone addiction and anxiety is not statistically significant. This conclusion challenges the homogenized assumption of equating depression and anxiety (43), and suggests that researchers should distinguish the distinct mechanisms underlying different negative emotions when exploring their influencing factors.

Third, this study verifies the negative effects of preference for solitude within the Chinese collectivistic cultural context. Unlike findings from Western studies which propose that moderate solitude benefits mental health (44), the current research demonstrates that a high level of preference for solitude amplifies the adverse impacts of mobile phone addiction on friendship quality and depressive symptoms. This result highlights the essential role of cultural factors in interpreting the associations between personality traits and mental health, and provides empirical evidence from China for cross-cultural research on preference for solitude.

### 4.4 Practical implications

In terms of practical implications, the findings offer three specific intervention directions for universities to address college students’ mobile phone addiction and emotional problems. Firstly, universities should attach importance to the protective function of friendship quality. By organizing diverse campus group activities, club events and class activities, universities can encourage students to engage in more offline interpersonal interactions, build and maintain high-quality friendships, and thereby buffer the negative impacts of mobile phone addiction on emotional health (66). Secondly, special attention should be paid to students with a high preference for solitude, who constitute a high-risk group for depressive symptoms correlated with mobile phone addiction. University mental health practitioners are advised to deliver individualized interventions for this population, helping them strike a balance between solitude and social engagement, improve interpersonal skills, and engage in beneficial activities during solitary time rather than excessively using mobile phones. Thirdly, targeted education on rational mobile phone use should be carried out to guide students to use mobile phones appropriately and set clear boundaries for usage. This helps prevent them from indulging in the virtual world at the expense of real-life interpersonal relationships, and ultimately reduces the psychological risks associated with mobile phone addiction at the source.

## 5 Limitations and future recommendations

The present study has several notable limitations. First, this study used a cross-sectional research design. This design only reveals concurrent associations among variables and cannot support definitive causal conclusions. Future studies may employ longitudinal follow-up or experimental designs to further clarify the causal ordering and long-term mechanisms underlying the relationships among mobile phone addiction, friendship quality, preference for solitude, depression, and anxiety. Second, convenience sampling was used to recruit college student participants, and the sample exhibited an unbalanced gender distribution, which may limit the external validity and generalizability of the findings. Future research may adopt stratified random sampling and include participants from different regions, various types of higher education institutions, and specific student groups. Third, this study relied on self-reported data. Although the common method bias test did not indicate significant issues, the influence of social desirability bias may still exist. Follow-up studies could integrate multi-source measurement approaches and incorporate objective data on mobile phone use (e.g., usage duration and application types) to reduce subjective reporting bias and improve the reliability of the results. Finally, the proposed model focused on the mediating role of friendship quality and the moderating role of preference for solitude. Future research may further expand this framework by introducing relevant variables such as personality traits, family support, and campus adaptation, so as to enrich and refine the mechanism model linking mobile phone addiction to emotional health.

## 6 Conclusions

Among Chinese college students, our findings support the mediating role of friendship quality in the associations between mobile phone addiction and elevated depressive and anxiety symptoms. Additionally, preference for solitude functions as a significant moderating variable that amplifies the correlational patterns linking mobile phone addiction to reduced friendship quality and increased depressive symptoms. No significant moderating effect of preference for solitude was observed for the direct association between mobile phone addiction and anxiety. This study elaborates on the correlational and conditional patterns of problematic mobile phone use and negative emotions within a Chinese college student sample, providing nuanced, culture-specific empirical evidence for understanding youth emotional health issues in digital contexts.

## Data Availability

The raw data supporting the conclusions of this article will be made available by the authors, without undue reservation.
